# The past, present, and future of predator–prey interactions in a warming world: Using species distribution modeling to forecast ectotherm–endotherm niche overlap

**DOI:** 10.1002/ece3.11067

**Published:** 2024-03-01

**Authors:** Jessica L. Hill, Matthew Grisnik, Ryan J. Hanscom, Jeet Sukumaran, Timothy E. Higham, Rulon W. Clark

**Affiliations:** ^1^ Department of Biology San Diego State University San Diego California USA; ^2^ Department of Agricultural and Environmental Sciences Tennessee State University Nashville Tennessee USA; ^3^ Department of Biology Coastal Carolina University Conway South Carolina USA; ^4^ Department of Evolution, Ecology, and Organismal Biology University of California Riverside California USA

**Keywords:** climate change, ecological niche modeling, predator–prey interactions, rattlesnake, small mammal

## Abstract

Climate change has the potential to disrupt species interactions across global ecosystems. Ectotherm–endotherm interactions may be especially prone to this risk due to the possible mismatch between the species in physiological response and performance. However, few studies have examined how changing temperatures might differentially impact species' niches or available suitable habitat when they have very different modes of thermoregulation. An ideal system for studying this interaction is the predator–prey system. In this study, we used ecological niche modeling to characterize the niche overlap and examine biogeography in past and future climate conditions of prairie rattlesnakes (*Crotalus viridis*) and Ord's kangaroo rats (*Dipodomys ordii*), an endotherm–ectotherm pair typifying a predator–prey species interaction. Our models show a high niche overlap between these two species (*D* = 0.863 and *I* = 0.979) and further affirm similar paleoecological distributions during the last glacial maximum (LGM) and mid‐Holocene (MH). Under future climate change scenarios, we found that prairie rattlesnakes may experience a reduction in overall suitable habitat (RCP 2.6 = −1.82%, 4.5 = −4.62%, 8.5 = −7.34%), whereas Ord's kangaroo rats may experience an increase (RCP 2.6 = 9.8%, 4.5 = 11.71%, 8.5 = 8.37%). We found a shared trend of stable suitable habitat at northern latitudes but reduced suitability in southern portions of the range, and we propose future monitoring and conservation be focused on those areas. Overall, we demonstrate a biogeographic example of how interacting ectotherm–endotherm species may have mismatched responses under climate change scenarios and the models presented here can serve as a starting point for further investigation into the biogeography of these systems.

## Graphical abstract

In this study, we used ecological niche modeling to characterize the niche overlap and examine biogeography in past and future climate conditions of prairie rattlesnakes (*Crotalus viridis*) and Ord's kangaroo rats (*Dipodomys ordii*), an endotherm–ectotherm pair typifying a predator–prey species interaction. As temperatures rise, there is potentially greater risk of disruption in these systems due to a differing physiological response to changing conditions.

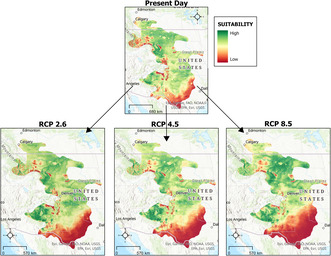

## 1 INTRODUCTION

Increasing anthropogenic carbon emissions are leading to warming temperatures across the globe and this is one of the leading factors driving biodiversity loss (Balint et al., 2011; Habibullah et al., 2021; Martay et al., 2017; Mooney et al., 2009). A large portion of conservation and research effort is focused on how endangered or threatened species are directly impacted by climate change, such as loss of appropriate suitable habitat, or an increase in physiologically intolerable conditions. However, climate change will also have indirect outcomes that warrant attention as well, including the disruption of ecosystem function through altered species interactions (Gilman et al., 2010). Species interactions that involve a keystone species or ecosystem engineer may have even stronger impacts due to the multitude of connections to other species within the community. Furthermore, climate change is expected to have the strongest impact on interactions that involve both an ectotherm and an endotherm due to the inherent physiological mismatch stemming from the fundamentally different way in which these species thermoregulate (Dell et al., 2014). Endotherms regulate their body temperature internally, whereas ectotherms' rely on external sources for heat in order to regulate their body temperature, activity, and performance (Gillooly et al., 2017). Perhaps, the most fundamental way in which these species interactions could be altered through climate change is from changes in habitat suitability stemming from increasing temperatures within their current geographic range (Fontúrbel et al., 2021). As a result of their mismatch, similar habitat conditions could result in differing suitability and therefore important biogeographical outcomes such as one member of the species pair becoming locally extinct in parts of their range. The distribution of suitable habitat in the past, as well as an examination of the niche of each species, could also provide additional context for how changing conditions could interact with the underlying physiological features of these species interactions.

**FIGURE 1:**
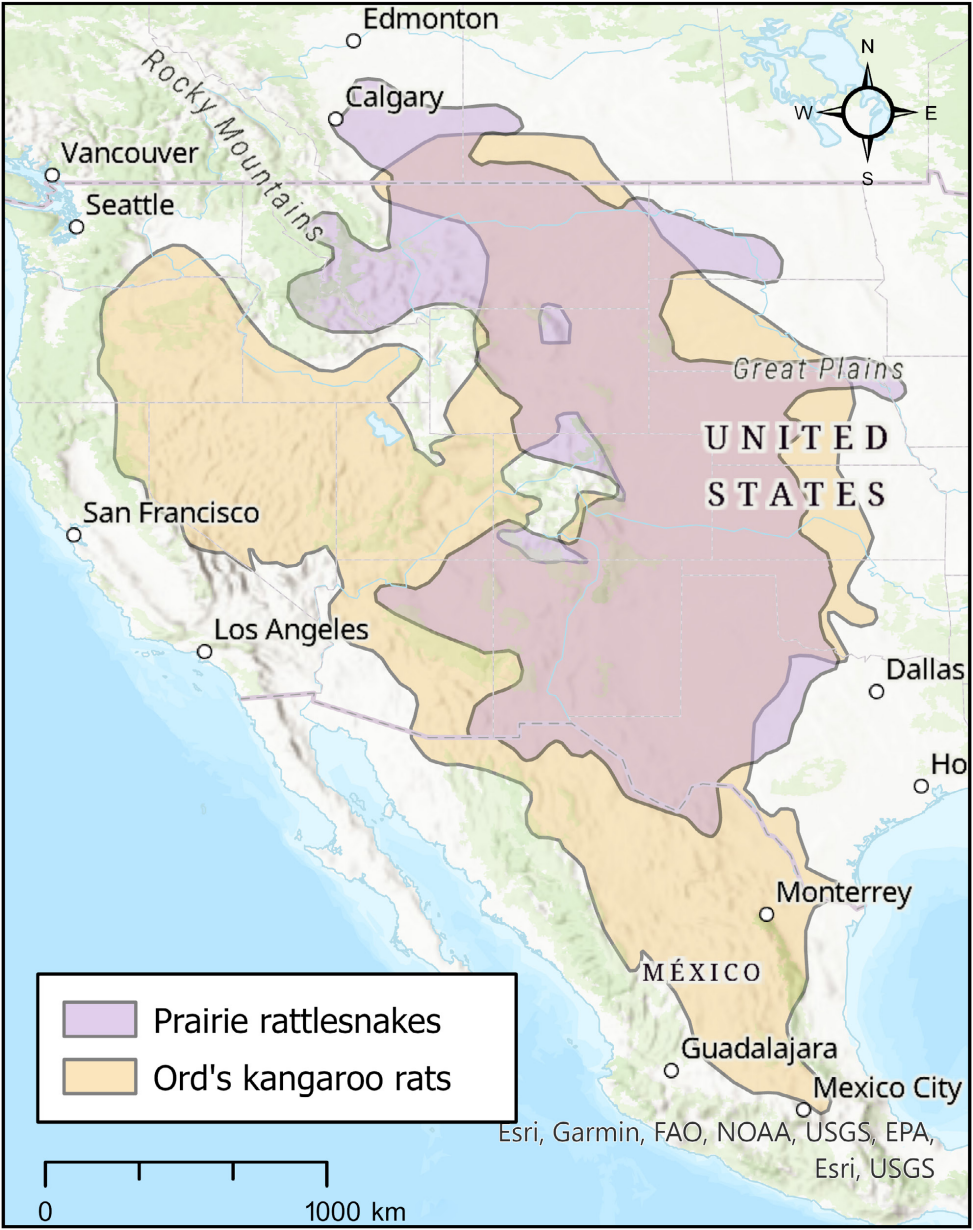
Range map of both species in our study (*Crotalus viridis* and *Dipodomys ordii*). Ranges also represent the study area for each ecological niche model created. Range map data sources: (https://www.iucnredlist.org/species/64339/12771847, https://www.iucnredlist.org/species/6691/115083268).

Here, we use the predator–prey species interaction between prairie rattlesnakes (*Crotalus viridis*) and Ord's kangaroo rats (*Dipodomys ordii*). The ranges of both animals encompass a wide latitudinal gradient with a large amount of environmental variation, which provides a strong foundation for studying the impacts of climate change, as responses to the outcomes may be significantly different across latitudes (Figure 1). In addition, both wide‐ranging species are abundant where they occur, providing key linkages within their ecosystem. Ord's kangaroo rats are especially well established as ecosystem engineers and as a keystone species (Heske et al., 1993). This is due to their creation of complex burrow systems that are used by a variety of other species and their role as obligate granivores that move and cache a wide variety of plant seeds (Bowers et al., 1987; Kerley et al., 1997). Prairie rattlesnakes then exert a top‐down influence on Ord's kangaroo rats via predation, as Ord's kangaroo rats are frequently the most common prey item in the diet of these small mammal specialists (Holycross, 1993; Laundre et al., 2010; Rothe‐Groleau, 2022). Despite the ecological importance of these species and their interaction, the dynamics of their biogeography has not been studied in concert over broad spatial and temporal scales.

Although many models of both snake and small mammal species have been constructed to examine the shifts in habitat suitability for individual species or regional groups of taxa (Baltensperger & Huettmann, 2015; Louppe et al., 2019; Lourenço‐de‐Moraes et al., 2019; Piquet et al., 2021), only a few studies apply the same modeling framework to species linked in a predator–prey interaction (Holt et al., 2018; Zahoor et al., 2023). Utilizing the same framework is important when determining how co‐occurrence of the two species may change, as variation in the selection of algorithms and environmental variables can impact model results. It is therefore difficult to make direct comparisons between different studies (Allouche et al., 2006; Qiao et al., 2019). Despite this, individual models of similar taxa may provide insight into the potential temporal biogeography of our study system. Studies that created ecological niche models (ENMs) of snake species suggest both increases and decreases in suitable habitat under climate change conditions and there does not seem to be a consensus among ectothermic reptiles (González‐Fernández et al., 2018; Kalboussi & Achour, 2018; Kirk et al., 2021; Kurnaz, 2023; Lourenço‐de‐Moraes et al., 2019; Piquet et al., 2021; Saptoka et al., 2021). Similarly, models of small mammal distributions suggest different responses to climate change depending on the species and modeling method (Baltensperger & Huettmann, 2015; Mathewson et al., 2017; Morueta‐Holme et al., 2010; Riddell et al., 2021). For example, a study performed on 17 small mammal species within Alaska found that northern groups of species would experience habitat loss under climate change scenarios, whereas the distribution of southern groups would increase (Baltensperger & Huettmann, 2015). Overall, ENMs may be difficult to extrapolate, even among taxa with similar natural histories, underscoring the importance of using the same modeling scheme to examine how suitable habitat may shift for a predator and prey species interaction.

In addition to understanding the biogeography of this ectotherm–endotherm interaction, there are benefits of directly examining and comparing their niches and environmental requirements for occurrence. It is often difficult to determine the degree of importance of biotic interactions on the distribution of a species' suitable habitat, and calculating niche overlap can be an alternative method to direct integration in models (Warren et al., 2008). This method has been used previously to examine overlap between predator and prey (Holt et al., 2018) and host–parasite species pairs (De Vivo & Huang, 2022). Additionally, overlap metrics have been used recently to examine similarities in ecological factors such as space use and foraging habits, as climate change leads to species colonizing new areas (Berlusconi et al., 2022; Chen et al., 2022). Understanding niche overlap in these scenarios can help inform competition and threats to local wildlife. Overall, examining the niche overlap of a species interaction may reveal the relative importance of abiotic factors or biotic factors in driving co‐occurrence.

We used ENMs—also referred to as species distribution models (SDMs)—to investigate the distribution of suitable habitat and niche overlap within predator–prey species interaction between prairie rattlesnakes (*Crotalus viridis*) and Ord's kangaroo rats (*Dipodomys ordii*). ENMs are increasingly popular for understanding how species ranges may change in the near future, as well as the degree to which those ranges overlapped in the past. Here, we created ENMs for prairie rattlesnakes and Ord's kangaroo rats using the same modeling framework to examine how suitable habitat availability has changed over time and may be influenced by climate change. Furthermore, we examined the overlap between these two species niches and the change in suitable habitat over their area of co‐occurrence.

## 2 METHODS

### 2.1 Study area

This study was conducted across the Great Plain's region of North America (Figure 1). For each individual species ENM, we used the IUCN range map as a baseline for the area of the model (Cassola, 2016; Frost et al., 2007). Each shapefile was downloaded and then buffered to a biologically relevant distance based on the dispersal ability and home range of the species. For prairie rattlesnakes, we chose a buffer of 1.5 km based on home‐range data from Bauder et al. (2015), and for Ord's kangaroo rats, we chose a buffer of 36 m based on the largest shifts in center of activity (COA) found for a closely related species, *Dipodomys merriami* (Behrends et al., 1986). We used IUCN range maps and did not incorporate larger surrounding areas because our focus was on areas where each species can be found currently or could realistically disperse to in near‐term future scenarios.

### 2.2 Occurrence data

We downloaded occurrence records for both prairie rattlesnakes and Ord's kangaroo rats from GBIF and VertNet using R (R Core Team, 2023). GBIF, the Global Biodiversity Information Facility, is a public database that includes occurrence records on most taxa globally. VertNet is a similar database of global biodiversity data containing geographic records from numerous taxa. Our downloaded point databases were cleaned by removing all incomplete records (missing key information, such as latitude or longitude), removing all records that were of subspecies, and removing duplicates. Records that were in the NAD27 projection were converted into the WGS84 projection. We also thinned records by removing those within 5 km of each other by placing a temporary 5 km × 5 km grid over the points and removing those that were too close together, which helps reduce the bias caused by over‐sampling in well‐known locations (Kramer‐Schadt et al., 2013). This step‐by‐step cleaning process was completed for each species and resulted in 2728 and 1960 occurrence records of prairie rattlesnakes and Ord's kangaroo rats, respectively.

We generated 10,000 “pseudo‐absences” to serve as the background for both of our models, as models perform better when spatial bias found in the occurrence dataset is replicated in the pseudo‐absences (Hertzog et al., 2014; Kramer‐Schadt et al., 2013; Phillips et al., 2009). We selected 10,000 pseudo‐absences because it has been shown previously that maxent performs best with this amount of background points (Barbet‐Massin et al., 2012). We accomplished this by using a target‐specific approach (Phillips et al., 2009) where we created a bias file to represent sampling effort. To create this file, we downloaded occurrence records of similar species from the GBIF database. For the prairie rattlesnake model, this was all snake species that occurred throughout their range and for the Ord's kangaroo rat model, this was a suite of small mammal genera that also occur throughout their range. We then used this bias file to sample the 10,000 points throughout the ranges of the target species while accounting for sampling effort.

### 2.3 Environmental data

We initially defined 22 environmental predictors for use in building our ENMs. This included the 19 bioclimatic predictors (1970–2000) in the WorldClim 1.4 database (Hijmans et al., 2005) as well as elevation, terrain ruggedness index (TRI), and topsoil sand content. We included elevation due to the large variation of this metric throughout both species' ranges. The elevation layers for both species were GTOPO30 tiles which we downloaded from the USGS Earth Explorer (USGS, 2000). We then merged tiles together using QGIS. We also included terrain ruggedness index as increased ruggedness has been shown to increase microhabitat variation and therefore areas available for snake refuge (Kirk et al., 2021). TRI was calculated from the previously downloaded elevation layer using the terrain ruggedness raster analysis function in QGIS. Lastly, we included topsoil sand content (percent sand) because Ord's kangaroo rats are more abundant in areas with sparsely vegetated, sandy soils where they can construct their burrow systems (Gummer, 1997; Kissner et al., 2009). Topsoil sand content was downloaded from the Unified North American Soil Map (Liu et al., 2014). All environmental layers were at a 30 arc second (1 km) resolution, projected to WGS84, and were masked to the buffered range of the appropriate species for each model. We performed a Pearson's correlation analysis to remove highly correlated variables (≥0.8) (Castellanos et al., 2019). We chose which variable to keep based on our own knowledge of the ecology of each species and the ecological relevance of the set of variables. This resulted in a total of 13 bioclimatic variables as well as the three additional layers of elevation, TRI, and percent sand, for a total 16 environmental layers input into each model (Table 1). We used the same layers for both species to allow for direct comparisons.

**TABLE 1:** Environmental data sources.

Data	Model type	Resolution	Emission scenario	Source
13 BCV (C)	–	30 s	–	https://www.worldclim.org/data/v1.4/worldclim14.html
13 BCV (2070)	1. BCC‐CSM1‐1 2. GFDL‐CM3 3. MIROC5	30 s	RCP 2.6, 4.5, 8.5	https://www.worldclim.org/data/v1.4/cmip5.html
13 BCV (MH)	1. CCSM4 2. MIROC‐ESM 3. MPI‐ESM‐P	30 s	–	https://www.worldclim.org/data/v1.4/paleo1.4.html
13 BCV (LGM)	1. CCSM4 2. MIROC‐ESM 3. MPI‐ESM‐P	2.5 min	–	https://www.worldclim.org/data/v1.4/paleo1.4.html
Elevation	–	30 s	–	https://www.usgs.gov/centers/eros/science/usgs‐eros‐archive‐digital‐elevation‐global‐30‐arc‐second‐elevation‐gtopo30
Sand Fraction	–	30 s	–	https://daac.ornl.gov/cgi‐bin/dsviewer.pl?ds_id=1242
TRI	–	30 s	–	N/A. Derived from elevation

*Note*: Adapted from Table 2 from Zahoor et al. (2023).

Abbreviations: BCV (2070), bioclimatic variables for 2070; BCV (C), bioclimatic variables for current time; BCV (LGM), bioclimatic variables for the last glacial maximum; BCV (MH), bioclimatic variables for the mid‐Holocene; RCP, representative concentration pathway; TRI, terrain ruggedness index.

### 2.4 Modeling framework

We chose the Maximum Entropy (MaxEnt) modeling algorithm to create ENMs for both prairie rattlesnakes and Ord's kangaroo rats (Elith et al., 2011; Phillips et al., 2006) as we have a presence‐background scheme of occurrences and this method has been shown to outperform other machine learning methods (Elith et al., 2006). We ran MaxEnt using R Version 2023.06.2 + 561 to create both models with five replicates using cross‐validation and used marginal response curves, jackknife test, and percent contribution to examine contributions and relationships of individual layers. We also adjusted the regularization multiplier (rm) for each model separately using the *ENMeval* package by testing rm values from 0.5 to 3 in 0.5 increments (Kass et al., 2021). In order to avoid over or under‐fitting each model, the regularization multiplier with the lowest delta AICc (Warren & Seifert, 2011) was chosen, resulting in a rm of 1 for the prairie rattlesnake model (the default) and a rm of 0 for the Ord's kangaroo rat model. Both models were evaluated using the area under the receiver operating characteristic curve (AUC). AUC is a popular threshold‐independent evaluation metric for ENMs and measures the ability of the model to distinguish between species presence and absence (Hanley & McNeil, 1982). The value of AUC ranges from 0 to 1, with the median value of 0.5 represent a random chance of predicting presence or absence.

### 2.5 Future and past predictions

We predicted the future distributions of prairie rattlesnakes and Ord's kangaroo rats with expected changes due to climate change using representative concentration pathways (RCPs), which are projected greenhouse gas emission scenarios into the year 2100 and represent the radiative forcing of greenhouse gases on future climate change (van Vuuren et al., 2011). We chose three different RCPs from a recent IPCC assessment (IPCC, 2014): RCP 2.6 (an optimistic scenario), RCP 4.5 (a mid‐range scenario), and RCP 8.5 (a worst case, “business as usual” scenario) (van Vuuren et al., 2011). All climate data were downloaded from the WorldClim 1.4 database (Hijmans et al., 2005) because historical climate data from WorldClim 2.1 are not yet available. All future predictions were completed for the year 2070 (average: 2061–2080). Three global circulation models (GCMs) were selected, BCC‐CSM1‐1, GFDL‐CM3, and MIROC5 in an effort to capture variation between different models based on the model genealogy of CMIP5 developed by Knutti et al. (2013). We then averaged the three GCMs within each RCP.

We predicted the past distribution of both species for the Mid‐Holocene (MH, ~6000 years ago) and the Last Glacial Maximum (LGM, ~22,000 years ago). Three GCMs were also chosen for past predictions, CCSM4, MIROC‐ESM, and MPI‐ESM‐P, as these are the most widely used GCMs for the LGM projections. We then averaged the three GCMs for each time period (MH and LGM). All future and past climate data were downloaded from the WorldClim 1.4 database. The environmental layers of elevation, TRI, and percent sand were held constant in all past and future distribution predictions as data does not exist that models these variables in different time periods and we do not expect substantial variation.

### 2.6 Abiotic niche overlap

We calculated the abiotic niche overlap of prairie rattlesnakes and Ord's kangaroo rats using both Schoener's *D* (Schoener, 1968) and Warren's *I* (Warren et al., 2008). These are both metrics of niche overlap based on environmental variables that vary from 0 (no overlap) to 1 (complete overlap). These metrics use the environmental variables included in our MaxEnt models, meaning that these measures of niche overlap only encompass the selected abiotic factors that may be shaping a species niche. It is common to use both metrics due to the fact that *D* has been used for much longer (and thus can be used for direct comparisons to older literature) and involves a simpler calculation but includes a biological assumption that the probability of occupancy within a cell actually represents species density whereas *I* is a newer metric that does not carry this assumption (Warren et al., 2008). We used the function *nicheOverlap* in the package *dismo* in R to perform both calculations (Hijmans et al., 2017).

### 2.7 Changes in suitability (vulnerability assessment)

We performed a vulnerability assessment to examine changes in suitable habitat over time for both species individually and the change of suitable habitat in areas of co‐occurrence without using a threshold to distinguish suitability. We did not use thresholding because when using presence‐background data, MaxEnt scores do not indicate probability of presence and thus cannot be interpreted as prevalence (Almeida et al., 2022; Guillera‐Arroita et al., 2015). Following others (Leão et al., 2021; Rose et al., 2023), changes in habitat were calculated as percentages as follows for individual species:

$$\text{Current to Future suitability}=\frac{\left(H_{f}-H_{c}\right)}{H_{c}}$$

$$\text{Past to Current}=\frac{\left(H_{c}-H_{p}\right)}{H_{p}}$$

where *H* = sum of suitability values in the appropriate raster layer, *c* = current, *f* = future, and *p* = past. We used these two formulas to calculate a percent change in available suitable habitat for each species from current to future time periods (under climate change) and from past to current time periods.

The change in suitable habitat in areas of co‐occurrence was calculated by first defining the area of overlap using the *st_intersection()* function in the package *sf* (Pebesma, 2018) in R. After establishing this separate area, we calculated the combined changes in suitable habitat for both species together as follows:

$$\text{Current to Future}=\frac{\left(\left({\mathrm{HP}}_{f}+{\mathrm{HO}}_{f}\right)-\left({\mathrm{HP}}_{c}+{\mathrm{HO}}_{c}\right)\right)}{\left({\mathrm{HP}}_{c}+{\mathrm{HO}}_{c}\right)}$$

$$\text{Past to Current}=\frac{\left(\left({\mathrm{HP}}_{c}+{\mathrm{HO}}_{c}\right)-\left({\mathrm{HP}}_{p}+{\mathrm{HO}}_{p}\right)\right)}{\left({\mathrm{HP}}_{p}+{\mathrm{HO}}_{p}\right)}$$

where HP = suitability of prairie rattlesnakes and HO = suitability of Ord's kangaroo rats.

All the above analyses were performed under each of the three future climate RCPs (2.6, 4.5, and 8.5) and each of the two past time periods (MH and LGM).

## 3 RESULTS

### 3.1 Abiotic niche overlap

Using 16 environmental variables and the MaxEnt modeling algorithm, the performance of both models was fair (Prairie rattlesnakes AUC = 0.679 [Appendix 1A], [Ord's kangaroo rats AUC = 0.725] [Appendix 1B]). These performance metrics are in line with expectations for models created over such a large area and with many occurrence points, as these factors have been shown to reduce AUC values (Boria & Blois, 2018). Both niche overlap metrics we calculated indicated that these two species have highly similar abiotic niches (*D* = 0.863 and *I* = 0.979). Some degree of ecological similarity was also indicated by the percent contribution of each environmental variable from MaxEnt (Appendix 1A,B), as both species had an approximately 30% contribution of temperature seasonality (Prairie rattlesnakes = 29.3%, Ord's kangaroo rats = 33.5%). However, the rankings of environmental variables demonstrate that there are still some differences in the species' niches despite their high overlap. First, while both had some contribution of annual mean temperature, they differed drastically in their actual influence (Prairie rattlesnakes = 33.4%, Ord's kangaroo rats = 6.7%). Additionally, the ranking of the four most important variables for prairie rattlesnakes was annual mean temperature (33.4%), temperature seasonality (29.3%), mean temperature of warmest quarter (11.5%), and precipitation of warmest quarter (8.0%). The ranking of the four most important variables for Ord's kangaroo rats was temperature seasonality (33.5%), ruggedness index (18.5%), percent sand (9.0%), and annual mean temperature (6.7%).

### 3.2 Changes in suitable habitat

**FIGURE 2:**
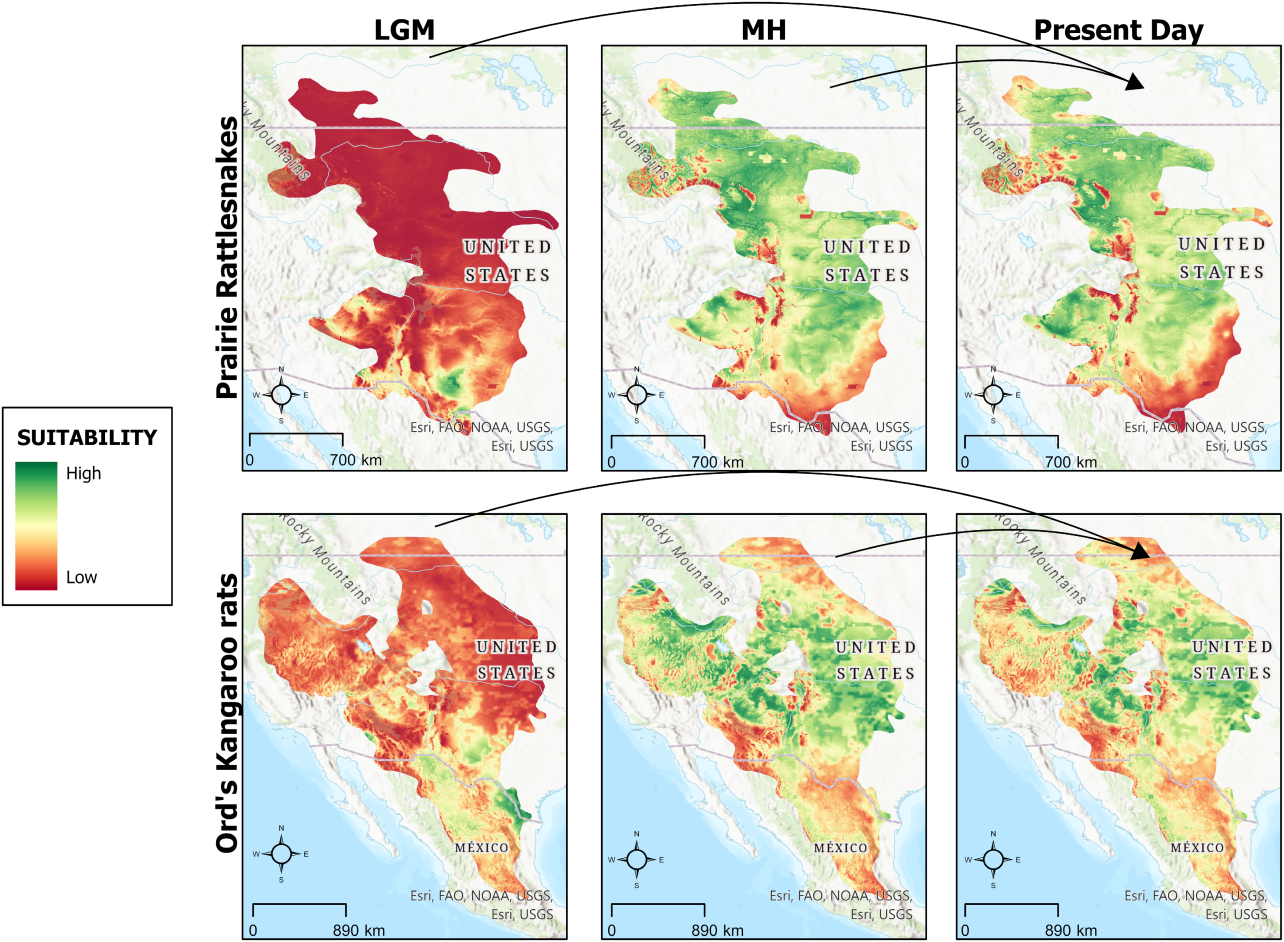
Change in habitat suitability for Prairie rattlesnakes and Ord's kangaroo rats from the last glacial maximum (LGM) and mid‐Holocene (MH).

We projected ecological niche models into the past (MH and LGM) and future under climate change scenarios (RCP 2.6, 4.5, and 8.5) to examine how the availability of suitable habitat may shift over a wide temporal scale. The past distributions of suitable habitat (Figure 2) qualitatively indicate that both prairie rattlesnakes and Ord's kangaroo rats may have experienced a southern refugia during the last glacial maximum (~22,000 years ago), as indicated by small areas with high habitat suitability in the southern end of both of their ranges (Figure 2). Following this time period, by the mid‐Holocene (~6000 years ago), both species had experienced a large expansion of suitable habitat, with mid‐Holocene habitat suitability closely resembling present day (Figure 2).

**FIGURE 3:**
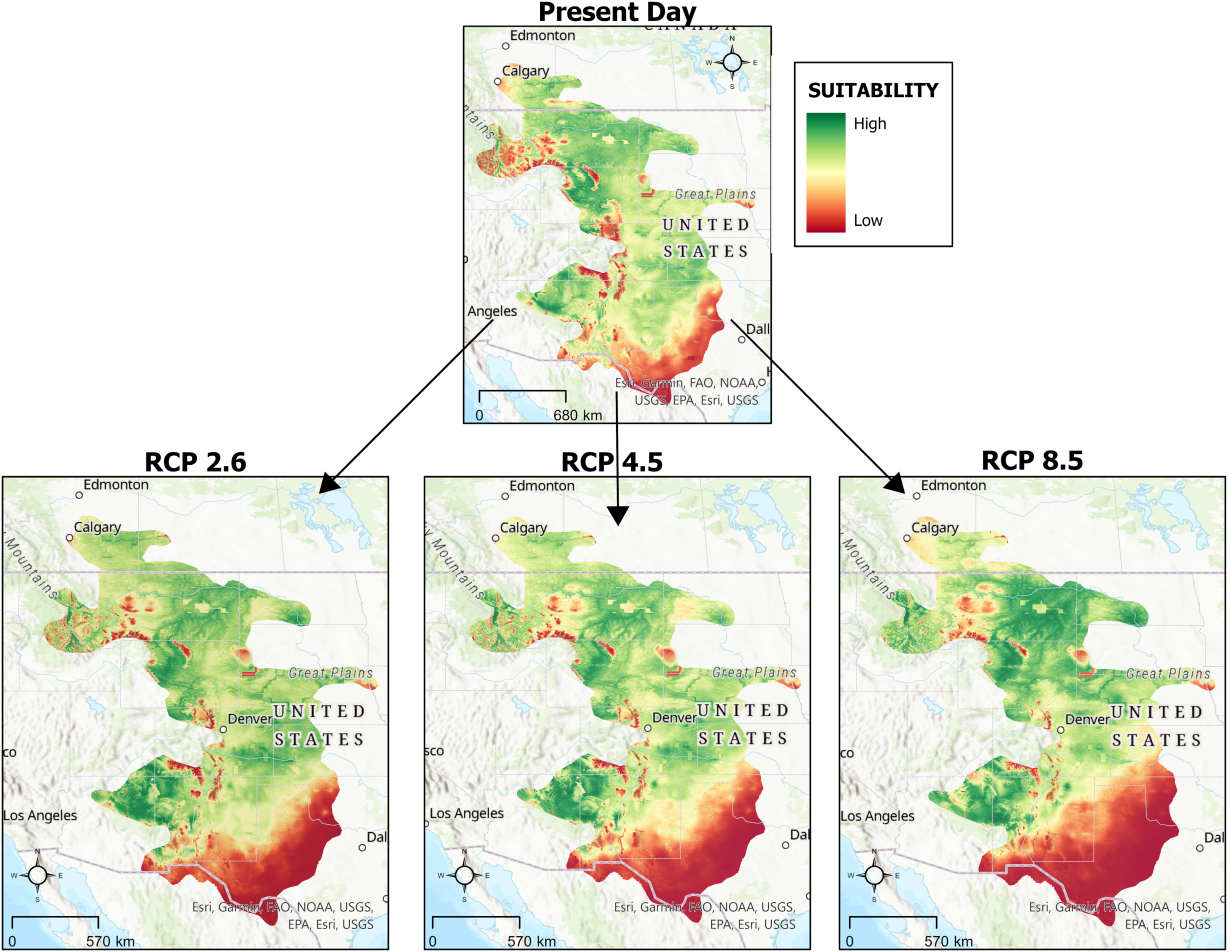
Change in habitat suitability for prairie rattlesnakes under three different representative concentration pathways (RCPs).

**FIGURE 4:**
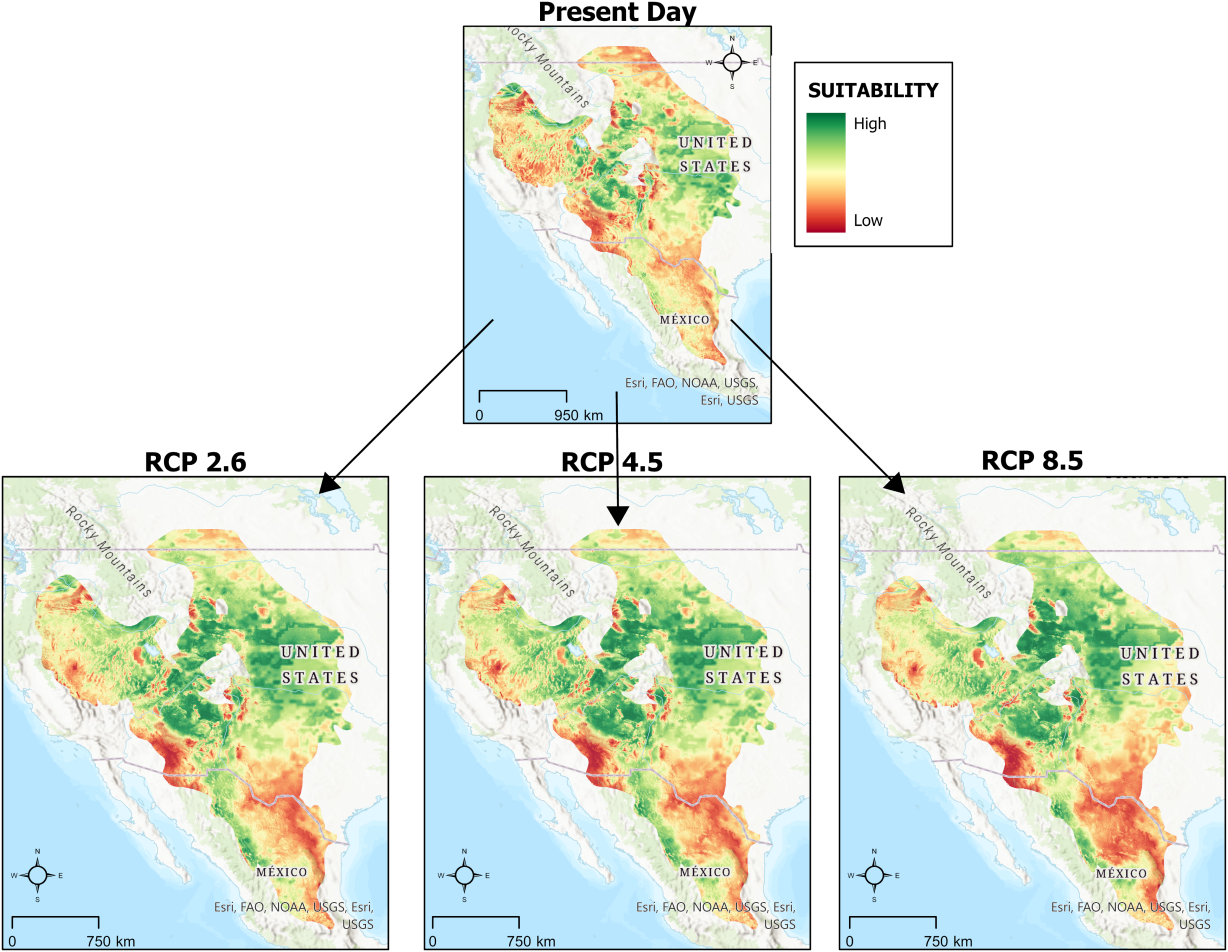
Change in habitat suitability for Ord's kangaroo rats under three different representative concentration pathways (RCPs).

Future projections under climate change scenarios indicate that both species may experience shifts in the locations of generally suitable habitat (Figures 3 and 4). For prairie rattlesnakes, our models estimate that habitat suitability will be reduced in the southern portion of their range, with some areas reaching suitability values of zero (Figure 3). For Ord's kangaroo rats, there is an increase in suitability in the northern extent of their range (Figure 4).

**FIGURE 5:**
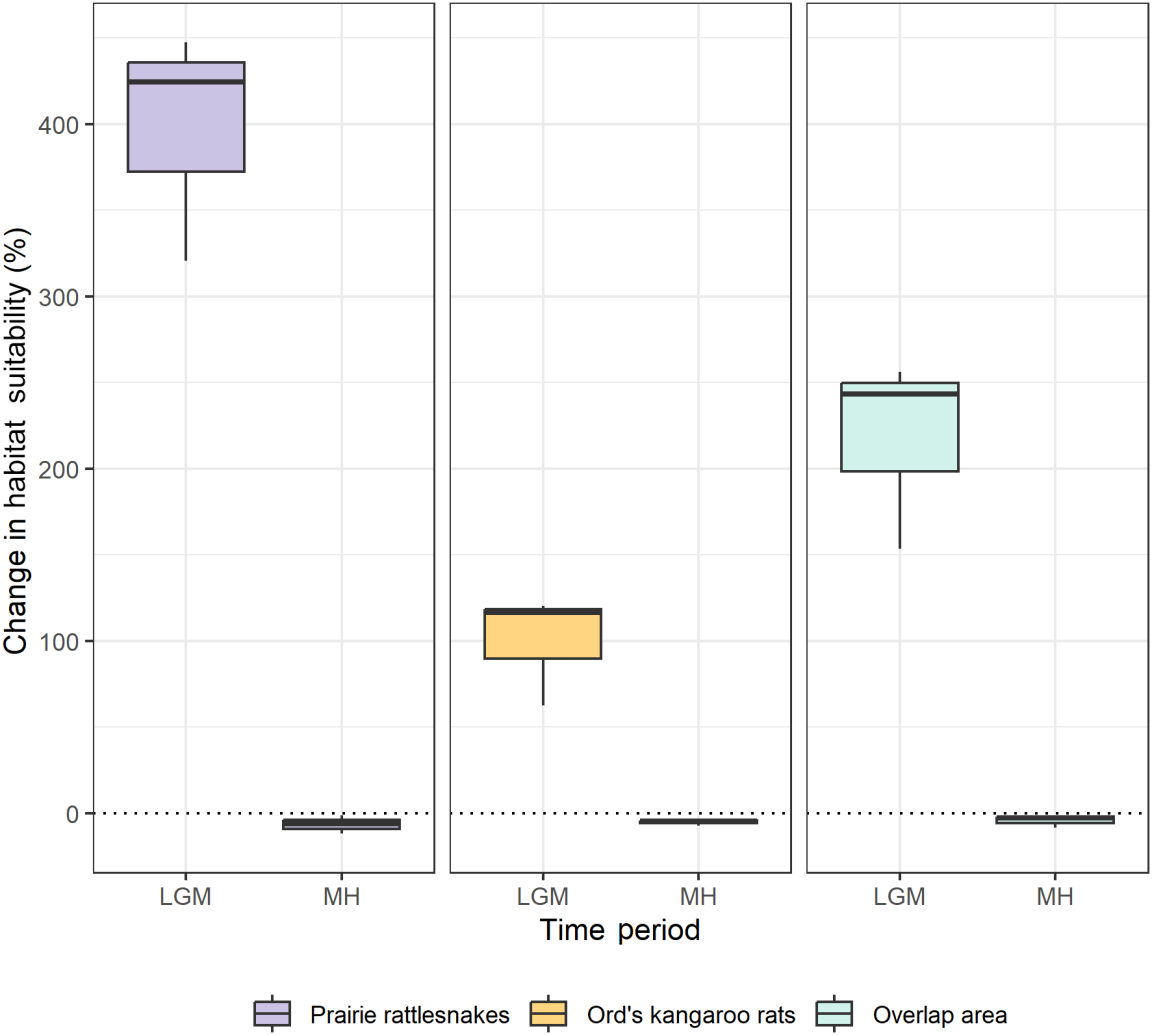
Boxplot showing the percent change in habitat suitability from paleoclimate scenarios to this day. Center lines represent the mean of the three GCMs.

**FIGURE 6:**
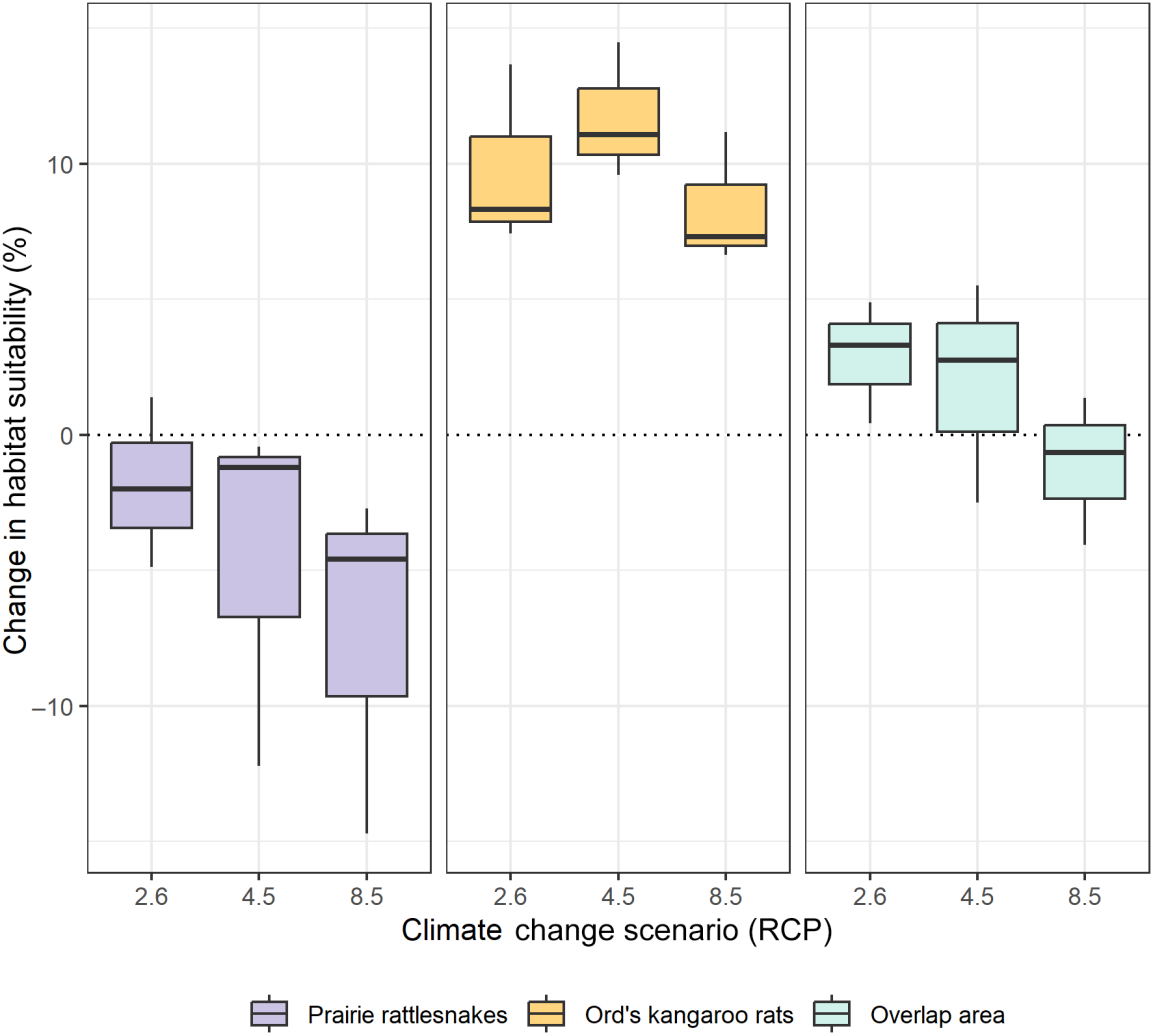
Boxplot showing the percent change in habitat suitability from present day to future climate change scenarios. Center lines represent the means of the three GCMs.

To quantify these changes, we performed a vulnerability assessment to examine the percent changes in overall suitable habitat (Table 2). These values are not spatially explicit but rather quantify change across the entire area. Prairie rattlesnakes experienced a 6.74% reduction in suitable habitat from the mid‐Holocene to this day, and a 396% increase from the last glacial maximum to this day (Figure 5, Table 2). Ord's kangaroo rats experienced a 5.27% reduction in suitable habitat from the mid‐Holocene to this day and a 95.88% increase in suitable habitat from the last glacial maximum to this day (Figure 5, Table 2). When we calculated changes for future scenarios, we found that prairie rattlesnakes were projected to experience a loss of suitable habitat under all future climate scenarios (RCP 2.6 = −1.82%, 4.5 = −4.62%, 8.5 = −7.34%, Figure 6). By contrast, Ord's kangaroo rats were projected to experience an increase in suitable habitat under all future climate change scenarios (RCP 2.6 = 9.8%, 4.5 = 11.71%, 8.5 = 8.37%, Figure 6).

**TABLE 2:** Results of vulnerability assessment—percent change in suitable habitat relative to this day.

	Climate change scenario (RCP)			Paleoclimate	
	2.6	4.5	8.5	LGM	MH
Prairie rattlesnakes	−1.82%	−4.62%	−7.34%	396.00%	−6.74%
Ord's kangaroo rats	9.80%	11.71%	8.37%	95.88%	−5.27%
Overlap area	2.87%	1.92%	−1.11%	210.00%	−4.40%

Additionally, we calculated the change in suitable habitat over the geographic area where the two species overlap. From the LGM to this day, there was a predicted 210% increase in shared suitable habitat, which is consistent with individual species' patterns (Figure 5). From the MH to current, there was a predicted slight decrease of 4.4% in shared suitable habitat. For each of the three climate change scenarios, there was a predicted slight increase in shared suitable habitat (RCP 2.6 = 2.87%, 4.5 = 1.92%, 8.5 = 1.11%, Figure 6).

## 4 DISCUSSION

We developed two ENMs representing an ectotherm–endotherm predator–prey species pair to examine the changes in suitable habitat over time and the degree to which their abiotic niche overlap may change under future warming scenarios. When examining the distribution of suitable habitat in the past, we saw patterns of a southern refugia in the LGM followed by increased suitable habitat during the MH for both species. When projecting our models into future climate change scenarios, we found a slight decrease in available habitat for prairie rattlesnakes but an increase for Ord's kangaroo rats. There was an overall pattern of suitability loss in the southern portions of both species' ranges. Additionally, we found high overlap between the species' abiotic niches and our models predicted overall stability in shared suitable habitat under future climate change scenarios.

### 4.1 Past suitability (LGM and MH)

Our models of the past habitat suitability of these two species suggests that they both experienced a southern refugia during the LGM and subsequently expanded during the MH. The severe reduction in suitable habitat during the LGM is consistent with a previous study involving prairie rattlesnakes which suggested that they had a reduced range during this time (Lawing & Polly, 2011). Furthermore, this refugia existing east of the Rocky Mountains provides confirmation of the phylogenetic suggestion that there was a split in the *Crotalus viridis* clade at this barrier (Pook et al., 2000). For Ord's kangaroo rats, their less pronounced reduction in habitat covers similar areas to those suggested by an ecological niche model of other members of the genus *Dipodomys* and suggests that some physiological and/or behavioral process allowed Ord's kangaroo rats to expand over a wider area than other members of the genus, such as *D. merriami* and *D. deserti* (Jezkova et al., 2015). The rapid expansion of suitable habitat we found for both species is consistent with the general concept that the rapid period of warming following the LGM resulted in range shifts for many taxa globally (Hewitt, 2000). However, we found that some of this expansion of available suitable habitat has already been lost from the MH to this day, potentially due to the early impacts of anthropogenic activities.

### 4.2 Future suitability resulting from climate change

As the environment changes due to climate change, prairie rattlesnakes are predicted to experience a loss of suitable habitat, whereas Ord's kangaroo rats may experience a slight expansion. Given the idea that there will be a future mismatch in the responses of ectotherms and endotherms under climate change (Dell et al., 2014), the abundance of prairie rattlesnakes may decrease as a result of decreasing suitability values in southern parts of their range. By contrast, Ord's kangaroo rats may not be as sensitive to these changes. As a result, there may be some loss of top‐down control in areas of decreased habitat suitability.

However, these species may not continue or cease to occupy certain areas in direct agreement with suitability values. Prairie rattlesnakes in southern portions of their range may experience increased physiological stress because of decreasing habitat suitability but continue to persist due to their demonstrated resiliency to rising temperatures (Crowell et al., 2021). They may also take advantage of favorable microhabitats that were averaged out due to the small resolution of our models. Additionally, Ord's kangaroo rats may not persist in the northern extent of their range, despite predicted increases in suitability, because existing populations in Canada are already classified as endangered due to habitat loss from anthropogenic development and other threats, such as increased vegetation stabilizing the sandy soils they prefer (COSEWIC, 2017). Although we do not suggest that our ENMs imply the presence or absence of these species, we demonstrate differences in the future suitability of their habitats and suggest that there may be differing responses leading to a potential disruption of this interaction.

This potential disruption can also be considered in the context of the vulnerability of prairie ecosystems more broadly. For example, a study performed on the prairie grass *A. gerardii* found that this tall grass species, which has extant populations in central areas of our system, may experience northeastward suitability shifts of 700 km or more, as well as a reduction in biomass in the current core distribution of the species (Smith et al., 2017). This grass is not distributed over the entirety of our focal system's range and may be demonstrative of some of the differences driving suitability loss in southern regions. Moreover, a study which created ENMs of 38 North American grassland bird species found that 42% of species were highly vulnerable to climate change (Wilsey et al., 2019). This illustrates that prairie and grassland ecosystems may remain under overall threat as a result of climate change—the possible disruption of the key predator–prey interaction we highlight here would be one of several potential factors negatively impacting this ecosystem.

### 4.3 Abiotic niche overlap and co‐occurrence

Despite the differing responses to climate change that we predict, the similarity in niche requirements, as indicated by high *D* and *I* values (*D* = 0.863 and *I* = 0.979), suggests that abiotic factors may contribute to both species being wide‐ranging and abundant. This is, in part, due to the fact that these metrics rely on the values of environmental variables within cells across the models (Warren et al., 2008). Furthermore, abiotic niche similarity may imply that both predator and prey could be vulnerable to similar anthropogenic or climatic changes. When a similar analysis was performed on the snow leopard and four of its prey species the main prey item, the Siberian ibex (*Capra sibirica*), was found to have the highest degree of overlap with the predator (Holt et al., 2018), which is consistent with our findings given that the Ord's kangaroo rat is the main prey item of prairie rattlesnakes (Holycross, 1993).

Despite the high degree of overlap, our models indicated that different environmental factors were top contributors in shaping the abiotic niche of each species. While important factors for prairie rattlesnakes included exclusively climate variables (such as temperature and precipitation), our model for Ord's kangaroo rats indicated that terrain ruggedness and percent sand were the second and third most important variables, respectively. It appears that rattlesnakes, as ectotherms, are more directly influenced by temperature, whereas Ord's kangaroo rats may rely more on the quality or type of available habitat, such as the occurrence of sandy soils that facilitate burrowing. These key differences in the species niches may contribute to the differing percent changes in suitable habitat we see under future climate change scenarios. While temperatures change in these scenarios, the terrain ruggedness and amount of sand in the soil is not expected to change on this time scale, which may explain in part why Ord's kangaroo rats experience an increase in available suitable habitat whereas prairie rattlesnakes do not, despite their overall high niche overlap.

When considering these differences, we also examined the partial dependence plots from the MaxEnt output (Appendix 1A,B) to make qualitative inferences about trends in the relationship between suitability and each important variable. For prairie rattlesnakes, we noticed an optimum temperature range that was mild and an increase in suitability with seasonality that asymptotes. For Ord's kangaroo rats, we noticed an increase in suitability with increased seasonality, a jump in suitability at high percentages of sand, and increasing suitability with increasing ruggedness. Generally, trends in the partial dependence plots are in line with our ecological expectations for both species, in terms of both environmental requirements and conservation needs.

Performing a separate calculation for the area of co‐occurrence when doing the vulnerability assessment demonstrates that, for the predator–prey system overall, suitable habitat will remain available. It appears that, assuming populations do not suffer losses from other external factors, there will not be a range‐wide rapid loss of prey for the rattlesnakes or loss of top‐down control on the kangaroo rats. However, the species' different environmental requirements may indicate a differing physiological response to temperature change and highlight unsuitable areas of the range where local conservation efforts could be developed to monitor the status of the ectothermic prairie rattlesnake more closely.

## 5 CONCLUSIONS

Overall, our study aimed to better understand an ectotherm–endotherm predator–prey system by examining the distribution of suitable habitat over a wide temporal scale and the niche overlap of the two species. Our findings further affirm paleoecological distributions and proposed phylogenetic divergence events of prairie rattlesnakes and Ord's kangaroo rats. Furthermore, future projections of suitable habitat demonstrate the role of biogeographic approaches for understanding the potential disruption of ectotherm–endotherm species interactions under climate change conditions. Additionally, we show that prairie rattlesnakes and Ord's kangaroo rats have highly similar abiotic niches and propose that they will continue to co‐occur and coevolve in most areas of their range where natural (i.e., not anthropogenically developed) habitat remains available.

Despite this, our models demonstrate that potential conservation attention may be warranted for populations in southern portions of the range of these species, where they may both experience a reduction in habitat suitability under future climate conditions. We also suggest a focus on identifying and conserving dispersal corridors to allow individuals to move between localities as needed in areas of potential stress due to threats to the ecosystem overall. Generally, increased monitoring of these species at the local level would allow for the detection of any disruptions to this interaction, which is a critical component of shortgrass prairie ecosystems. We also suggest further investigation into the niches of this interaction based on the differences in important environmental variables shown in the individual ENMs. Future questions could utilize the occurrence of the prey, Ord's kangaroo rats, as a predictor of prairie rattlesnake presence and vice versa. Furthermore, our models provide a foundation for creating more complex ecological niche models (e.g., joint distribution models) of this system and others like it to examine the geographic and environmental dynamics of biotic interactions.

## AUTHOR CONTRIBUTIONS

**Jessica L. Hill:** Conceptualization (equal); data curation (lead); formal analysis (equal); methodology (equal); writing – original draft (lead); writing – review and editing (lead). **Matthew Grisnik:** Conceptualization (equal); data curation (supporting); formal analysis (equal); methodology (equal); project administration (equal); writing – review and editing (supporting). **Ryan J. Hanscom:** Conceptualization (supporting); project administration (supporting); writing – review and editing (supporting). **Jeet Sukumaran:** Formal analysis (supporting); funding acquisition (equal); project administration (supporting); writing – review and editing (supporting). **Timothy E. Higham:** Funding acquisition (equal); project administration (supporting); writing – review and editing (supporting). **Rulon W. Clark:** Conceptualization (equal); funding acquisition (equal); project administration (equal); writing – original draft (supporting); writing – review and editing (supporting).

## CONFLICT OF INTEREST STATEMENT

The authors declare they have no competing interests.

### OPEN RESEARCH BADGES

This article has earned an Open Data badge for making publicly available the digitally‐shareable data necessary to reproduce the reported results. The data is available at https://doi.org/10.5061/dryad.h18931zsb.

## Data Availability

Cleaned occurrence records and environmental variables used for both models are available on Dryad (https://datadryad.org/stash/share/L7dm3orq7pllICjGHhvnjbro5vnqCRg45EJk5JC3YAg). Modeling procedures are reported following the ODMAP protocol (Zurell et al., 2020). Appendix 2. While not entirely bespoke, code is available on Zenodo per the ODMAP protocol's statement for code availability of SDMs (https://datadryad.org/stash/share/L7dm3orq7pllICjGHhvnjbro5vnqCRg45EJk5JC3YAg).

## APPENDIX 1

### A. Selected maxent output from *Crotalus Viridis* Model

Receiver operating characteristic (ROC) curve averaged over the replicate runs. The average AUC is 0.679 and the standard deviation is 0.005.

Table giving estimates of relative contributions of the environmental variables to the model.

Jackknife tests of variable importance using training gain, test gain, and AUC. Values shown are averages over replicate runs.

Partial dependency plots of each variable. Each curve represents a model creating only using the specific variable. These plots reflect the dependence of predicted suitability both on the selected variable and on dependencies induced by correlations between the selected variable and other variables. Variable values are on the *y* axis and suitability is on the *x* axis.

### B. Selected maxent output from *Dipodomys ordii* model

Receiver operating characteristic (ROC) curve averaged over the replicate runs. The average AUC is 0.725 and the standard deviation is 0.005.

Table giving estimates of relative contributions of the environmental variables to the model.

Jackknife tests of variable importance using training gain, test gain, and AUC. Values shown are averages over replicate runs.

Partial dependency plots of each variable. Each curve represents a model creating only using the specific variable. These plots reflect the dependence of predicted suitability both on the selected variable and on dependencies induced by correlations between the selected variable and other variables. Variable values are on the *y* axis and suitability is on the *x* axis.

## APPENDIX 2 ODMAP

**Table:**

**OVERVIEW**	
Authorship	*Authors*: Jessica Hill *Contact email*: jessicalyn132@gmail.com *Title*: The past, present, and future of predator–prey interactions in a warming world: Using species distribution modeling to forecast endotherm‐ectotherm niche overlap *DOI*: https://doi.org/10.1002/ece3.11067
Model objective	*SDM objective*: mapping/interpolation and forecasting/backcasting (transfer). These models also aimed to characterize the niche of the species *Target output*: Niche similarity values, habitat suitability, and changes in suitability over time
Taxon	Prairie rattlesnakes (*Crotalus viridis*) and Ord's kangaroo rat (*Dipodomys ordii*)
Location	Great plains region of North America
Scale of analyses	*Spatial extent*: Buffered IUCN ranges for both species *Spatial resolution*: 30 arc seconds (1 km) *Temporal resolution*: Models forecasted to both past (LGM and MH) and future (2070)
Biodiversity data overview	*Observation type*: Citizen science (GBIF and VertNet) *Response/Data type*: Presence‐only (pseudo‐absences generated)
Type of predictors	BIOCLIM predictors, elevation, terrain ruggedness index (TRI), and topsoil sand content
Conceptual model/Hypotheses	We aimed to characterize the similarity/dissimilarity of the niches of a predator–prey system as well as their geographic shifts in suitable habitat (suitability) from paleo‐time to future conditions under climate change
Assumptions	We assume that included ecological drivers shape the species niche. Certain predictors are assumed (held) constant throughout the time‐period due to data availability. We also assume species–environment equilibrium. Biases present in citizen science data were accounted for in the pseudo‐absence generation process
SDM algorithms	*Model algorithms*: MaxEnt. Chosen due to presence‐background sampling scheme and previously highlighted performance ability *Model complexity* *Model averaging*: Average of five replicates for each model
Model workflow	All variables were projected to WGS 84 and masked to the IUCN range of the species of each respective model. We then performed a Pearson's correlation analysis to remove highly correlated variables. We created present day models for each species and then forecasted each one into past and future time periods. Models were fitted by adjusting the regularization multiplier to result in the lowest AICc values. Models were evaluated using AUC and TSS
Software	*Software*: R version 4.1.1., QGIS for some environmental variable processing. *Code availability*: https://doi.org/10.5061/dryad.h18931zsb *Data availability*: https://doi.org/10.5061/dryad.h18931zsb
**DATA**	
Biodiversity data	*Taxon names*: *Crotalus viridis* and *Dipodomys ordii* *Taxonomic reference system*: Prairie rattlesnakes defined as Rafinesque, 1818. Does not include any pacific rattlesnakes, now defined as *Crotalus o. oreganus* and *Crotalus o. helleri*. Ord's kangaroo rats not delineated to subspecies and the disjunct Canadian population is included in analyses *Ecological level*: Species *Biodiversity data source*: Data were downloaded using R from GBIF and VertNet *Sampling design*: Citizen science data (N/A) *Sample size*: After cleaning, 2728 occurrence records of prairie rattlesnakes and 1960 occurrence records of Ord's kangaroo rats *Mask*: IUCN range of each species, buffered by 1.5 km for prairie rattlesnakes and 36 m for Ord's kangaroo rats *Absence data*: 10,000 pseudo‐absences were generated to represent the “background” using a target‐specific approach
Data partitioning	k‐fold cross validation
Predictor variables	*Predictor variables*: Climate: Annual mean temperature, annual precipitation, isothermality, mean diurnal range, mean temperature of driest quarter, mean temperature of warmest quarter, mean temperature of wettest quarter, precipitation seasonality, precipitation of coldest quarter, precipitation of driest month, precipitation of warmest quarter, precipitation of wettest month, and temperature seasonality. Others: topsoil sand fraction, terrain ruggedness index, and elevation *Data sources*: Climate data: Downloaded from WorldClim version 1.4 database. Elevation: Downloaded as GTOPO30 tiles from the USGS earth explorer and merged using QGIS. Terrain Ruggedness Index (TRI): TRI was calculated from the elevation layer. Topsoil Sand Content: Downloaded from the Unified North American Soil Map. (Available as an individual layer) *Spatial extent*: All predictors were masked to the buffered IUCN range of the species being modeled *Spatial resolution*: All data were downloaded at a 1 km resolution *Projection*: WGS 84, EPSG:4326 *Temporal extent*: Current Climate 1960–1990. Past Climate LGM (~22,000 years ago) and MH (~10,000 years ago). Future Climate 2070 (average of 2061–2080). No temporal information for topsoil sand fraction, elevation, or terrain ruggedness index *Data processing*: Terrain ruggedness index (TRI) was calculated from the elevation layer using the terrain ruggedness raster analysis function in QGIS
Transfer data for projection	*Future climate*: Future climate data were downloaded from the WorldClim 1.4 database for 2070. Data were averaged from three different global circulation models (GCM); BCC‐CSM1‐1, GFDL‐CM3, and MIROC5. Three emission scenarios were considered: RCP 2.6, 4.5, and 8.5. These represent low, middle, and high emissions *Past climate*: Downloaded from the WorldClim 1.4 database. Two time periods were included: the Last Glacial Maximum (LGM) and Mid‐Holocene (MH). Data were averaged from three GCMs; CCSM4, MIROC‐ESM, and MPI‐ESM‐P *Data processing*: Climate data for the LGM were resampled to 30 arc seconds from 2.5 arc minutes
**MODEL**	
Variable preselection	Initial variables were chosen based on the intent to model suitability through a large temporal scale (bioclim) and on ecological relevance to each species as indicated in their natural history. (Ruggedness influences rattlesnakes' ability to move through their environment, sandy soils influence kangaroo rats' ability to dig burrow systems)
Multicollinearity	A Pearson's correlation analysis was performed on an initial set of 22 predictor variables and eight were removed because of collinearity being high (>0.8). We removed one variable from each highly correlated pair
Model settings	*MaxEnt*: Default settings with tuning of the regularization multiplier based on AICc, resulting in an rm of 1 for prairie rattlesnakes and 0 for Ord's kangaroo rats. Five replicates with cross‐validation *Prediction*: Predictions were limited to the extent of the buffered IUCN ranges in order to represent feasible occurrence of individuals
Model estimates	We used marginal response curves, jackknife test, and percent contribution to examine variable importance
Model averaging/ensembles	Models were averaged from five replicates
Nonindependence	N/A
Threshold selection	We did not threshold out models as our aim was not to quantify potential presence of individuals. Rather, we performed a vulnerability assessment using suitability values (Leão et al., 2021, Rose et al., 2023)
**ASSESSMENT**	
Performance statistics	Models were evaluated using AUC (based on average of five replicates with cross‐validation) and true‐skills statistic (TSS) calculated manually using excel. TSS was calculated using the 10th percentile threshold
Plausibility checks	We examined response plots and visualized mapped predictions for both models. Two authors have also spent extensive time on the ground studying both organisms at three sites across their range
**PREDICTION**	
Prediction output	Predictions were created using climate data for paleo‐time and future climate scenarios (downloaded from WorldClim 1.4). We performed a vulnerability assessment to quantify and examine the change in suitability over this time‐period. We calculated this change for individual species ranges as well as specifically over their area od co‐occurrence. Outputs were represented as a percentage (e.g., A change of −7.34% decrease in suitable habitat for prairie rattlesnakes under RCP 8.5)
Uncertainty quantification	N/A
